# Whole-body MRI: detecting bone metastases from prostate cancer

**DOI:** 10.1007/s11604-021-01205-6

**Published:** 2021-10-25

**Authors:** Katsuyuki Nakanishi, Junichiro Tanaka, Yasuhiro Nakaya, Noboru Maeda, Atsuhiko Sakamoto, Akiko Nakayama, Hiroki Satomura, Mio Sakai, Koji Konishi, Yoshiyuki Yamamoto, Akira Nagahara, Kazuo Nishimura, Satoshi Takenaka, Noriyuki Tomiyama

**Affiliations:** 1 Department of Diagnostic and Interventional Radiology, Osaka International Cancer Institute, 3-1-69 Otemae, Chuo-ku, Osaka, 541-8567 Japan; 2 Department of Radiation Oncology, Osaka International Cancer Institute, 3-1-69, Otemae, Chuo-ku, Osaka, 541-8567 Japan; 3 Department of Urology, Osaka International Cancer Institute, 3-1-69, Otemae, Chuo-ku, Osaka, 541-8567 Japan; 4 Department of Orthopaedic Surgery, Osaka International Cancer Institute, 3-1-69, Otemae, Chuo-ku, Osaka, 541-8567 Japan; 5 Department of Diagnostic and Interventional Radiology, Osaka University Graduate School of Medicine, 2-2, Yamadaoka, Suita, Osaka, Suita, 565-0871 Japan

**Keywords:** Whole-body MRI, Prostate cancer, Bone metastases, DWIBS

## Abstract

Whole-body magnetic resonance imaging (WB-MRI) is currently used worldwide for detecting bone metastases from prostate cancer. The 5-year survival rate for prostate cancer is > 95%. However, an increase in survival time may increase the incidence of bone metastasis. Therefore, detecting bone metastases is of great clinical interest. Bone metastases are commonly located in the spine, pelvis, shoulder, and distal femur. Bone metastases from prostate cancer are well-known representatives of osteoblastic metastases. However, other types of bone metastases, such as mixed or inter-trabecular type, have also been detected using MRI. MRI does not involve radiation exposure and has good sensitivity and specificity for detecting bone metastases. WB-MRI has undergone gradual developments since the last century, and in 2004, Takahara et al., developed diffusion-weighted Imaging (DWI) with background body signal suppression (DWIBS). Since then, WB-MRI, including DWI, has continued to play an important role in detecting bone metastases and monitoring therapeutic effects. An imaging protocol that allows complete examination within approximately 30 min has been established. This review focuses on WB-MRI standardization and the automatic calculation of tumor total diffusion volume (tDV) and mean apparent diffusion coefficient (ADC) value. In the future, artificial intelligence (AI) will enable shorter imaging times and easier automatic segmentation.

## Introduction

There are significant advantages to using whole-body magnetic resonance imaging (WB-MRI) for detecting bone metastases. Evaluation can be performed with a single scan, which is potentially more cost-effective and time-saving for whole-body evaluations [1–5]. Moreover, it can be used for whole-body evaluation and treatment response monitoring. In particular, diffusion-weighted imaging (DWI) has become available for whole-body scanning, which has now been incorporated into the main sequence of WB-MRI. Prostate cancer is the most frequent malignant tumor in older men and is associated with a high rate of recurrence [6]. Therefore, the use of WB-MRI has become frequent.

This review article aims to provide a general statement on bone metastases, explain the developmental history of WB-MRI, provide an interpretation of imaging methods with WB-MRI, present the details of some typical cases of bone metastases from prostate cancer, and discuss the future of WB-MRI.

## General statement on bone metastases

Bone metastasis is a devastating condition that has wide-ranging negative impacts on the lives of patients with advanced cancer [7]. To date, no large-scale etiological studies on the prevalence or incidence of bone metastasis have been conducted worldwide [7]. However, the current 5-year survival rate for prostate cancer is > 95% across numerous countries [8], and an increase in survival time may increase the incidence of bone metastasis.

**Fig. 1:**
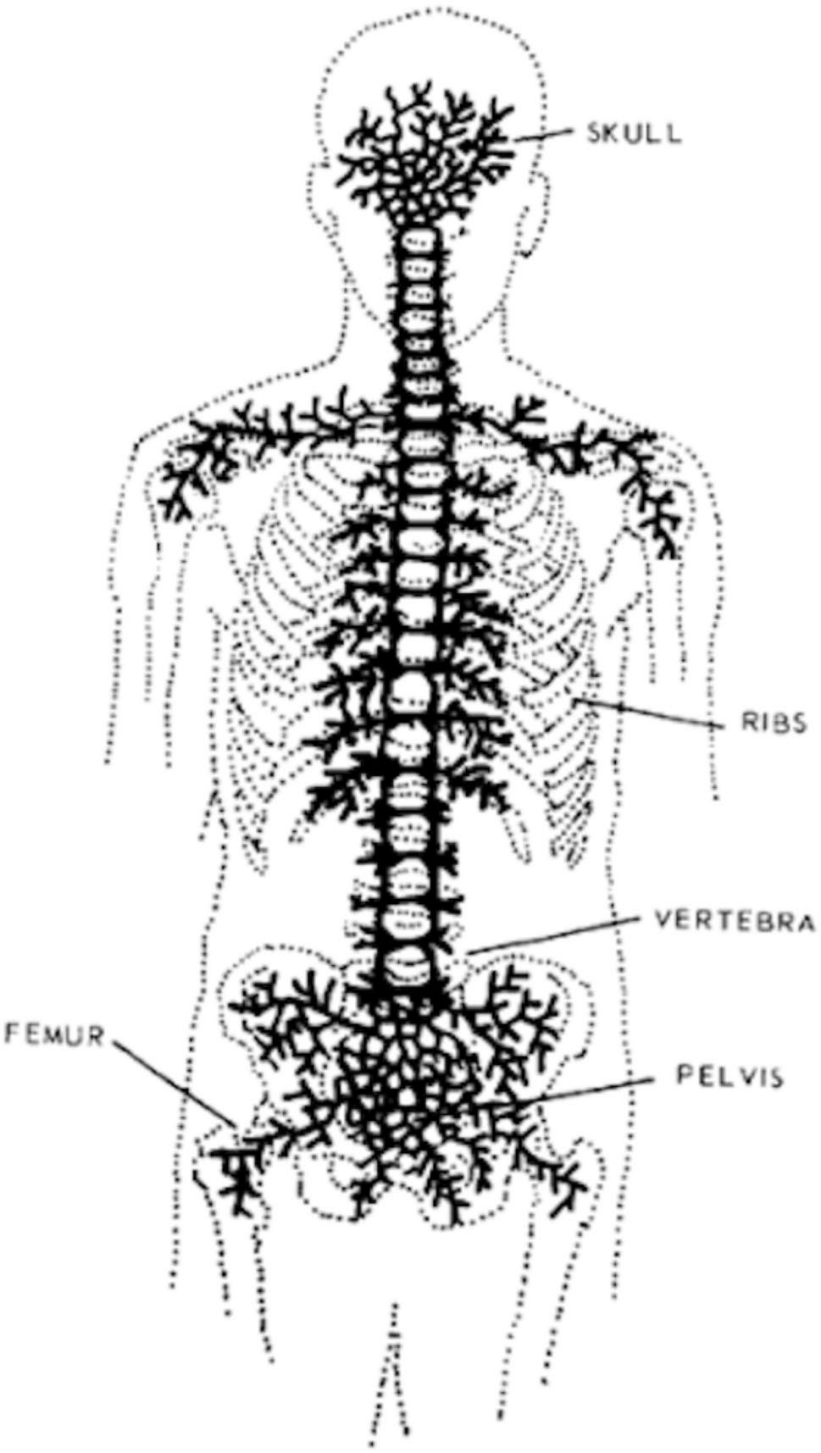
Batson’s venous plexus. Cited from *Diseases of the Spine and Spinal Cord* (Thomas N Byrne et al. P169, Oxford University Press)

Irrespective of the primary malignant location, bone metastases are commonly found in the spine, pelvis, shoulder, and distal femur [9, 10]. These bone lesions can cause serious complications, such as spinal cord and nerve root compression, pathological fracture, and hypercalcemia [9]. Bone metastases most commonly affect the axial skeleton. In adults, the axial skeleton contains red marrow, which suggests that the properties of the circulation, cells and extracellular matrix within this region assist in the formation of bone metastasis [10]. Batson [11] showed that venous blood from the breast and pelvis flowed not only into the vena cava but also into the vertebral venous plexus, which extends from the pelvis to throughout the epidural and peri-vertebral veins [11, 12] (Fig. 1). Blood drainage to the skeleton via the vertebral venous plexus may, at least in part, explain the tendency of breast and prostate cancers (as well as those arising in the kidney, thyroid, and lung) to produce metastases in the axial skeleton and limb girdles [10, 11].

Conventional classifications categorize bone metastases as osteoblastic, osteolytic and mixed types [9, 13]. This classification is based on the primary mechanism of interference with normal bone remodeling [13] and the uptake of radiotracers, which depends on the quantity of the calcification of the metastases and osteoblastic activity [9]. However, the recently identified inter-trabecular-type metastasis, which infiltrates the marrow space without altering the trabecular bone and is not radiologically visible but detectable on MRI, requires further characterization [14, 15].

**Fig. 2:**
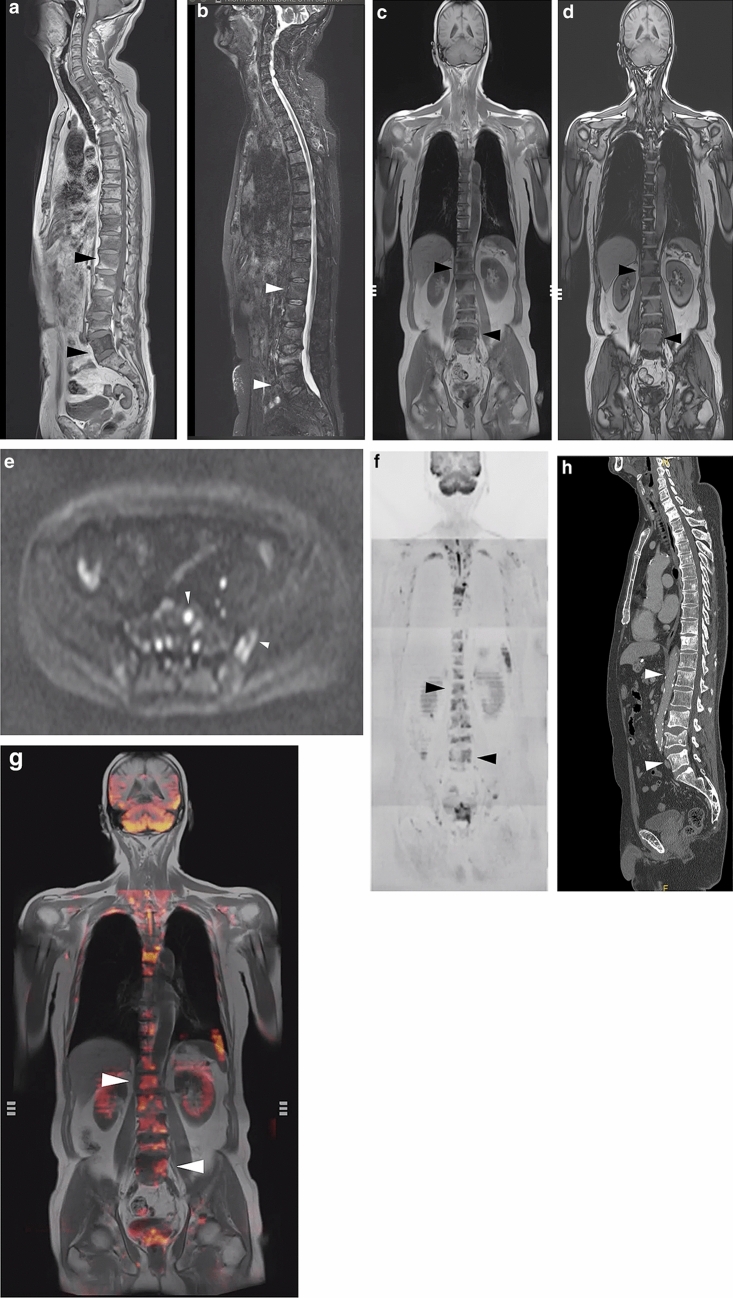
Sample case (**a**–**g**) and osteoblastic change (**h**). The figure presents a 69-year-old man with multiple bone metastases from prostate cancer. Serum PSA was 4.894 ng/mL. **a** Total spine T1W image. Multiple low-intensity areas are shown in the spine, including in the L1 and L5 (arrowheads). **b** Total spine STIR image. Multiple mild, high-intensity areas are shown in the spine, including in the L1 and L5 (white arrowheads). **c** Body coronal in-phase T1W image. Multiple low-intensity areas can be seen (arrowheads). **d** Body coronal out-of-phase T1W image. Multiple slight high-intensity areas are shown in the lumbar spine (arrowheads). **e** Axial *b* = 1000 of the DW image at the level of the pelvic bone. High-intensity areas are shown in the sacrum and left ilium (white arrowheads). **f** Coronal reconstructed DW image. This image is displayed as a black-and-white inverted image. Multiple high-intensity areas are shown in the spine, including in the L1 and L5 (arrowheads). **g** Fused image combining in-phase coronal T1W image with coronal reconstructed DW image. Multiple high-intensity areas are shown in the spine, including in the L1 and L5 (white arrowheads). CT was performed at approximately the same time and revealed osteoblastic metastases. **h** Sagittal reconstruction of the CT image. Multiple sclerotic lesions can be seen, including in the L1 and L5 (white arrowheads), which were correlated with the low-intensity area in the T1W image and the high-intensity area in DW image. The diagnosis was osteoblastic metastases

**Fig. 3:**
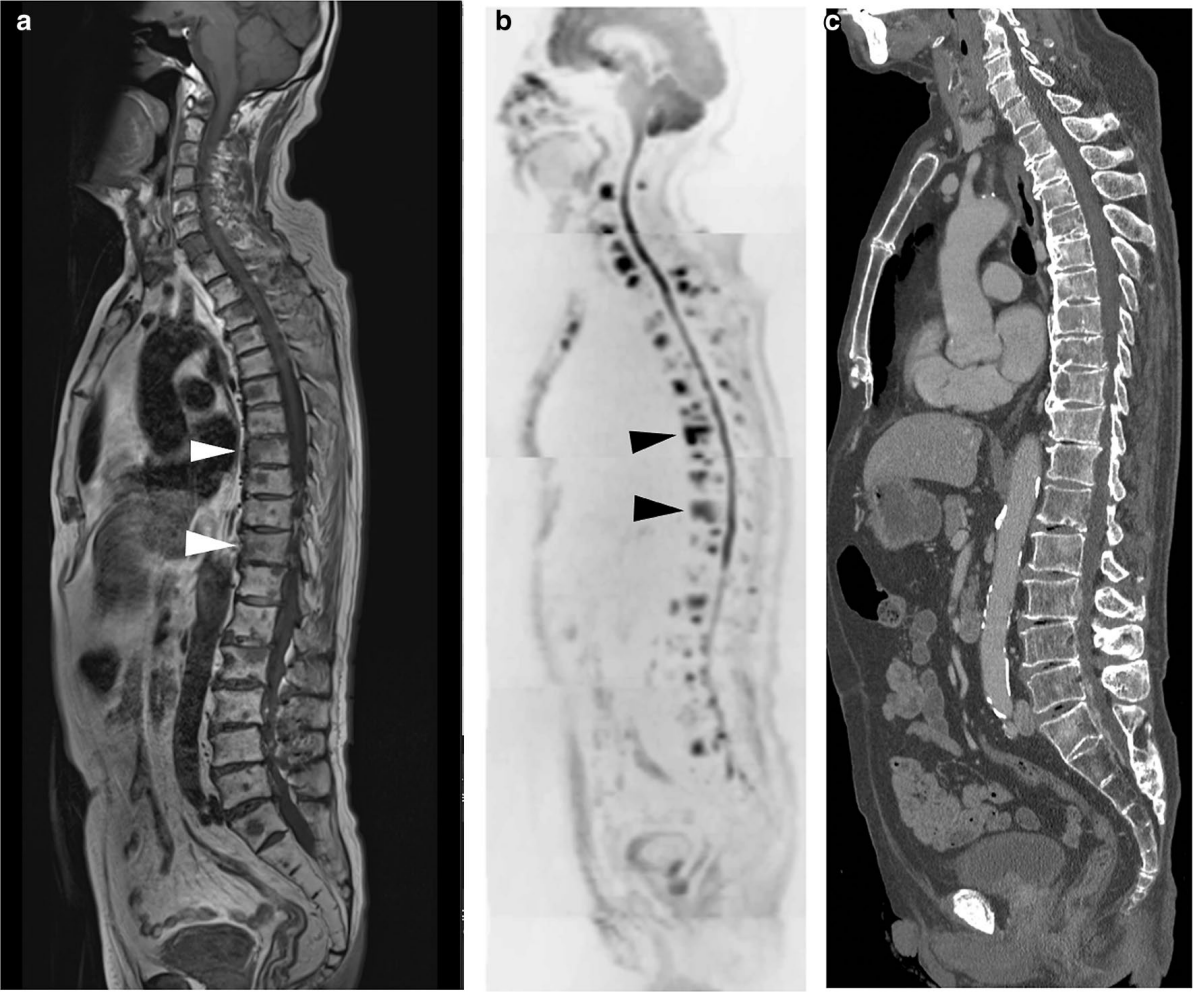
Mixed-type/intertrabecular metastases. A 78-year-old man with a 4-year history of prostate cancer and transition to castration-resistant prostate cancer (CRPC). Serum PSA was 5.954 ng/mL. **a** On T1W image, multiple low-intensity areas are shown, including in the Th8 and Th11 (white arrowheads). **b** On the DW sagittal reconstructed image, multiple high-intensity areas are shown, including in the Th8 and Th11(arrowheads). **c** On the CT reconstructed sagittal image, obvious sclerotic or lytic changes were not observed. In fact, the diagnosis of multiple bone metastases using only CT examination was not possible. These CT findings were defined as intertrabecular metastases

Bone metastases from prostate cancer are a well-established example of osteoblastic metastases [9, 13, 16] (Fig. 2). In prostate cancer, prostate-specific antigen (PSA) inhibits parathyroid hormone-related peptides, which leads to the enhancement of osteoblast function [9, 11, 17]. However, the development of various imaging modalities has enabled the detection of other types of metastases, such as mixed or inter-trabecular types (Fig. 3).

Imaging modalities for diagnosing bone metastases include technetium-99 m bone scintigraphy (BS), plain radiography, computed tomography (CT), MRI, and 18F fluorodeoxyglucose (FDG)–positron emission tomography (PET) [7, 13].

BS is highly sensitive but usually has low specificity [13]. It is more sensitive than plain film and CT scans; however, MRI is superior for the evaluation of vertebral metastases [18]. BS provides information on osteoblastic activity and skeletal vascularity with preferential uptake of tracers at sites with active bone formation, which reflects the metabolic reaction of bone during the disease process, whether neoplastic, traumatic or inflammatory [13, 19].

Although plain radiographs are highly specific, they have low sensitivity (44–50%) [13]. Because of limited contrast, medullary lesions are more difficult to detect in trabecular bone than in cortical bone [13, 18].

The sensitivity of CT for the diagnosis of bone metastases ranges from 71 to 100% [13, 20]. Bone destruction and sclerotic deposits are usually clearly shown, and any soft tissue extension of bone metastases can be easily visualized [13]. However, the ability to detect inter-trabecular spread remains controversial [21–24].

18F FDG-PET detects the presence of bone metastases by directly quantifying metabolic activity [7, 13].

MRI provides good contrast resolution of bone and soft tissue and therefore has good sensitivity and specificity for the detection for bone metastases [7, 9, 13]. However, limited field of view and long examination time pose problems, which existed even before the development of WB-MRI.

Traditionally, BS is the first choice for the diagnosis of bone metastases arising from prostate cancer. However, this method is considered insufficient, and a combination of CT and MRI is often used. Indeed, conventional modalities alone have been reported to be inadequate for the evaluation of treatment effects. [5, 25–27]. However, since the development of WB-MRI, the basis for diagnosing bone metastases from prostate cancer has changed.

## Developmental history of WB-MRI

Reports comparing BS with MRI for the detection of bone metastases have been available since the last century [18]. However, our research has indicated that the oldest reports that included the term “*whole-body* MRI” date back to 1997 [28, 29]. Since the beginning in the twenty-first century, various devices have been developed to enable a whole-body scan in a single session without the need to change the directions of the body, which include multichannel coil and table-top extenders [30–32].

In 2004, Takahara et al. [33] used DWI with background body signal suppression (DWIBS). Until the development of this method, DWI was predominantly used for investigations of the central nervous system, especially in cases of acute stroke [34]. However, several researchers have reported that various malignancies in the body show similar high signal intensities [33, 35–37].

Takahara et al. adapted DWI for whole-body malignancy screening using free- breathing, short T1 inversion recovery, and a high-resolution three-dimensional display. They demonstrated that free-breathing scans work effectively and that short T1 inversion recovery enables excellent fat suppression, which suggested that the method could be a powerful screening tool for malignances. In the field of WB-MRI, this paper was groundbreaking. Since then, numerous reports have been published on the addition of DWI to WB-MRI for the detection of bone metastases in not only prostate cancer, but also breast cancer, lung cancer, and multiple myeloma [5, 38–40]. Moreover, this led many researchers to pay more attention to the field of prostate cancer [6, 41–50].

There are numerous comparative studies on the use of BS with WB-MRI. However, because these studies relied on the use of various MR scanners and included various types of cases, the results are cross-sectional [25, 51–58]. However, in 2020, Sun et al. [27] performed a database search to conduct a meta-analysis to compare the diagnostic performance between WB-MRI and BS for the detection of bone metastases. The results showed that WB-MRI had higher but comparable patient-based specificity as BS (99% vs. 95%) but markedly higher sensitivity (94% vs. 80%). The authors concluded that WB-MRI has higher sensitivity and diagnostic accuracy than did BS and may be used for both the confirmation and exclusion of metastatic bone disease.

To date, many therapeutic agents have been developed [59–62], such as radium-223 dichloride [63] for castration-resistant prostate cancer (CRPC), which has a high occurrence of bone metastases [64]. With the increase in the number of treatment options and improvements in patient survival, the use of WB-MRI for providing accurate diagnosis and therapy monitoring has become crucial.

Since the mid-2010s, research has focused on the standardization [41–47] and therapy monitoring of WB-MRI [48].

**Fig. 4:**
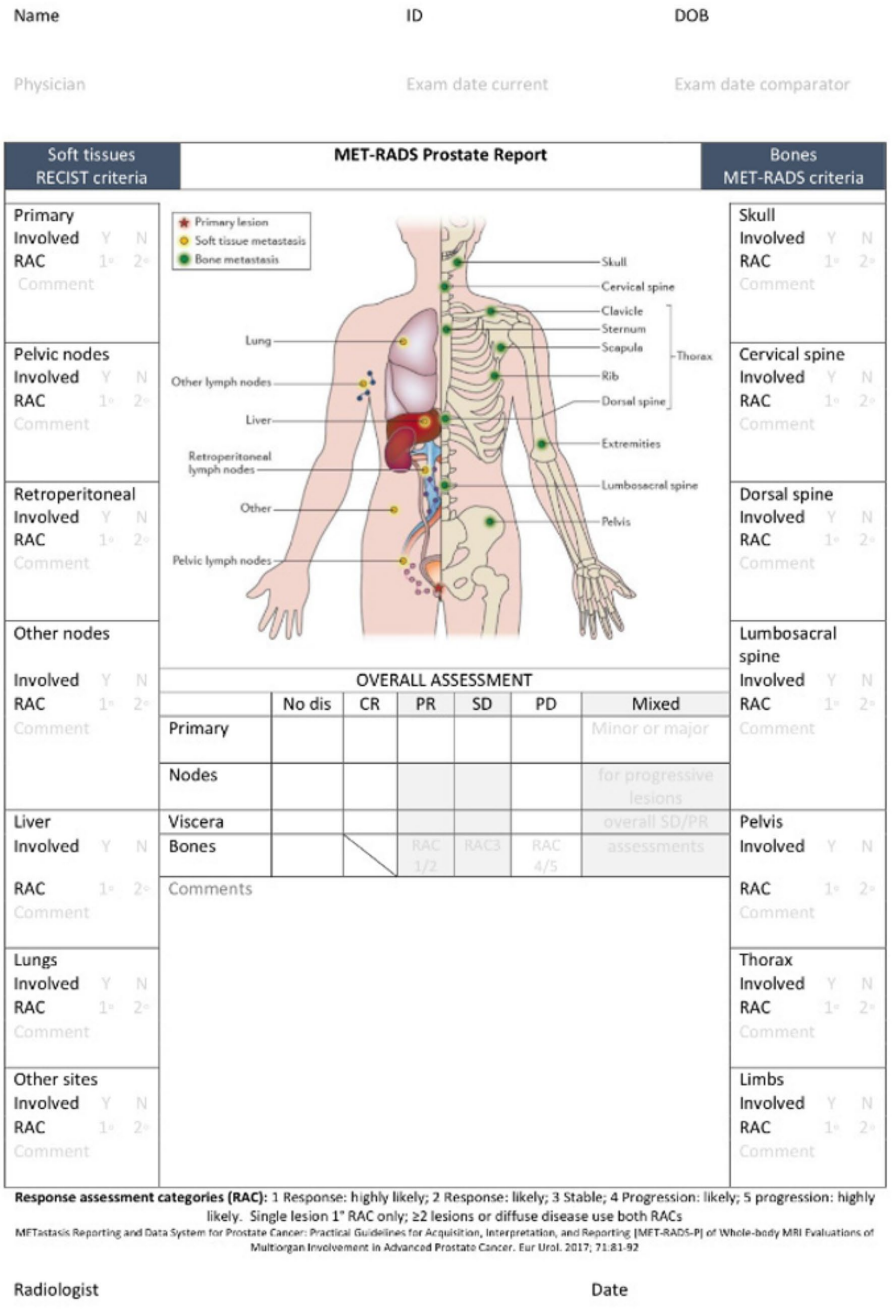
MET-RADS-P template [65]. The MET-RADS-P template form allocates the presence of unequivocally identified disease to 14 predefined regions of the body

Padhani et al. highlighted the need for expert recommendations for WB-MRI scans and developed the Metastasis Reporting and Data System for Prostate Cancer (MET-RADS-P) [41, 65]. An expert panel of the most experienced radiologists and nuclear medicine physicians in advanced prostate cancer imaging conducted a review [46] and formulated guidelines on the performance standards for WB-MRI for the assessment of multi-organ involvement in advanced prostate cancer (Fig. 4).

Padhani et al. [46] also reported the usefulness of WB-MRI for therapy monitoring. In addition, Padhani et al. suggested that WB-MRI provides a clear categorization of bone metastasis response and that the accurate assessment of therapy response would aid the rationale development of targeted therapies.

With progress of the standardization of WB-MRI and its use in therapy monitoring, there is now a need to quantify tumor volume and determine the apparent diffusion coefficient (ADC) value to enable precise evaluation of disease activity.

Blackledge et al. [66] reported the semi-automatic presentation of whole-body DWI for deriving tumor total diffusion volume (tDV) and associated global ADC.

This method is applicable for assessing treatment response in patients with bone metastases [47, 67–69]. Currently, the use of this device is favored for the precise evaluation of lesion detection and monitoring the therapeutic effect in patients with bone metastases.

## Interpretation of imaging methods with WB-MRI

### Recommended imaging methods and parameters

In this section, we show the representative imaging methods used in our department.

We used the Siemens Magnetom Prisma (3 T) scanner.

**Fig. 5:**
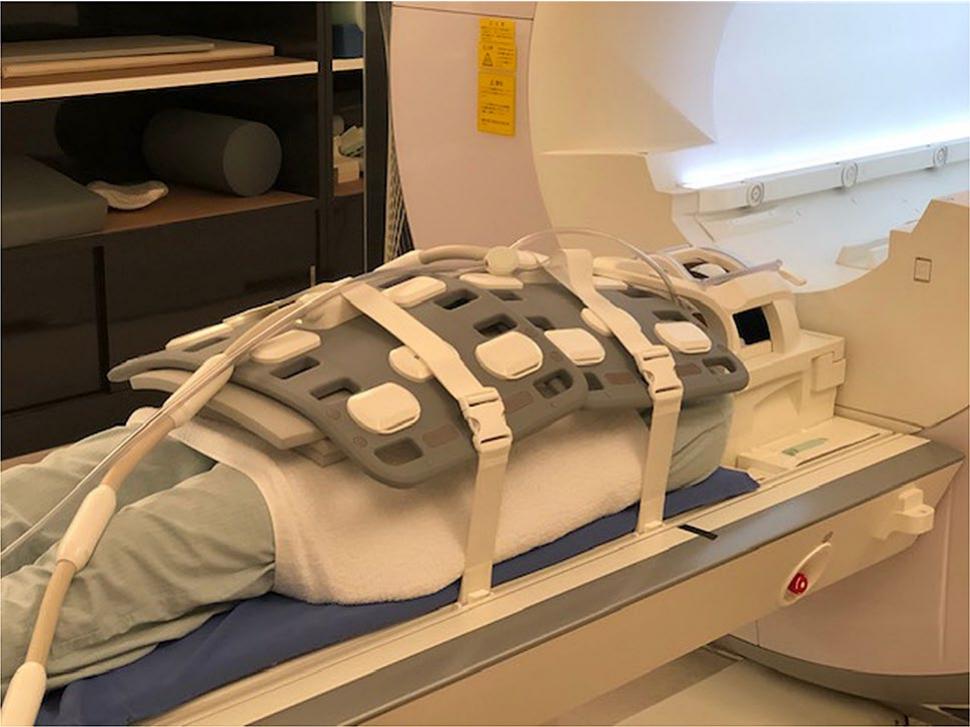
Multichannel coil: 20-channel head coil, 32-channel spine coil, and two or three 18-channel body-array coils were combined to cover the area of Batson’s venous plexus, which is an area with a predilection for bone metastases

Multiple matrix coils covered patients from the lower neck to the proximal femur (Fig. 5), which also covered the Batson’s venous plexus. Excluding the lower leg is controversial. In the trial by Lecouvet et al. [49], none of the patients had isolated peripheral metastases with WB-MRI, and only the axial-skeleton MRI in prostate cancer was missed.

The main examples of pulse sequences (Tables 1, 2, 3) and case (Fig. 2) are as follows:

**Table 1:** Total Spine sagittal sequences

	T1WI fast SE	STIR
TR/TE/TI (ms)	520/8.8	5000/87/230
Echo train length	2	14
Slice thickness (mm)	4/4.4(gap)	4/4.4(gap)
FOV of each station (mm)	420	420
Matrix	320 × 240	320 × 240
No. of stations	3	3
Time of each station	1 min 1 s	1 min 28 s
Other	19 slices	19 slices

**Table 2:** Body coronal sequences

TR/TE (ms)	3.93/1.32(opposed phase), 2.45 (in-phase)
Slice thickness (mm)	4
FOV of each station (mm)	450
Matrix	416 × 312
No. of stations	3
Time of each station (s)	17 s
others	Breath hold scan, flip angle 10°

**Table 3:** Axial DWI sequences

TR/TE/TI (ms)	6000/44/230
Slice thickness (mm)	5
FOV of each station (mm)	480
Matrix	256 × 192
No. of slices of each station	40
No. of stations	4 or 5
Time of each station	1 min 24 s
*b* value (mm^2^/s)	0 and 1000

#### Total spine sagittal sequences

T1-weighted imaging (T1WI) and short T1 inversion recovery (STIR) consist of three stations (Table 1, Fig. 2a and b).

#### Body coronal sequences

Dixon’s method consists of three stations (Table 2, Fig. 2c and d).

#### Axial diffusion-weighted sequences

Axial diffusion sequences are obtained with *b* value of 1000 (Fig. 2e) and 0, and the ADC map consists of four or five stations (Table 3).

The total examination time, including the positioning of the patient, is approximately 23 min.

After image acquisition, image processing is performed.

From the axial DWI images, coronal (Fig. 2f) and radial images are reconstructed and displayed as black-and-white reversed images. In-phase coronal T1WI- and DWI-coronal reconstructed images are fused, and the fusion images are reconstructed (Fig. 2g).

### Environment for image interpretation

**Fig. 6:**
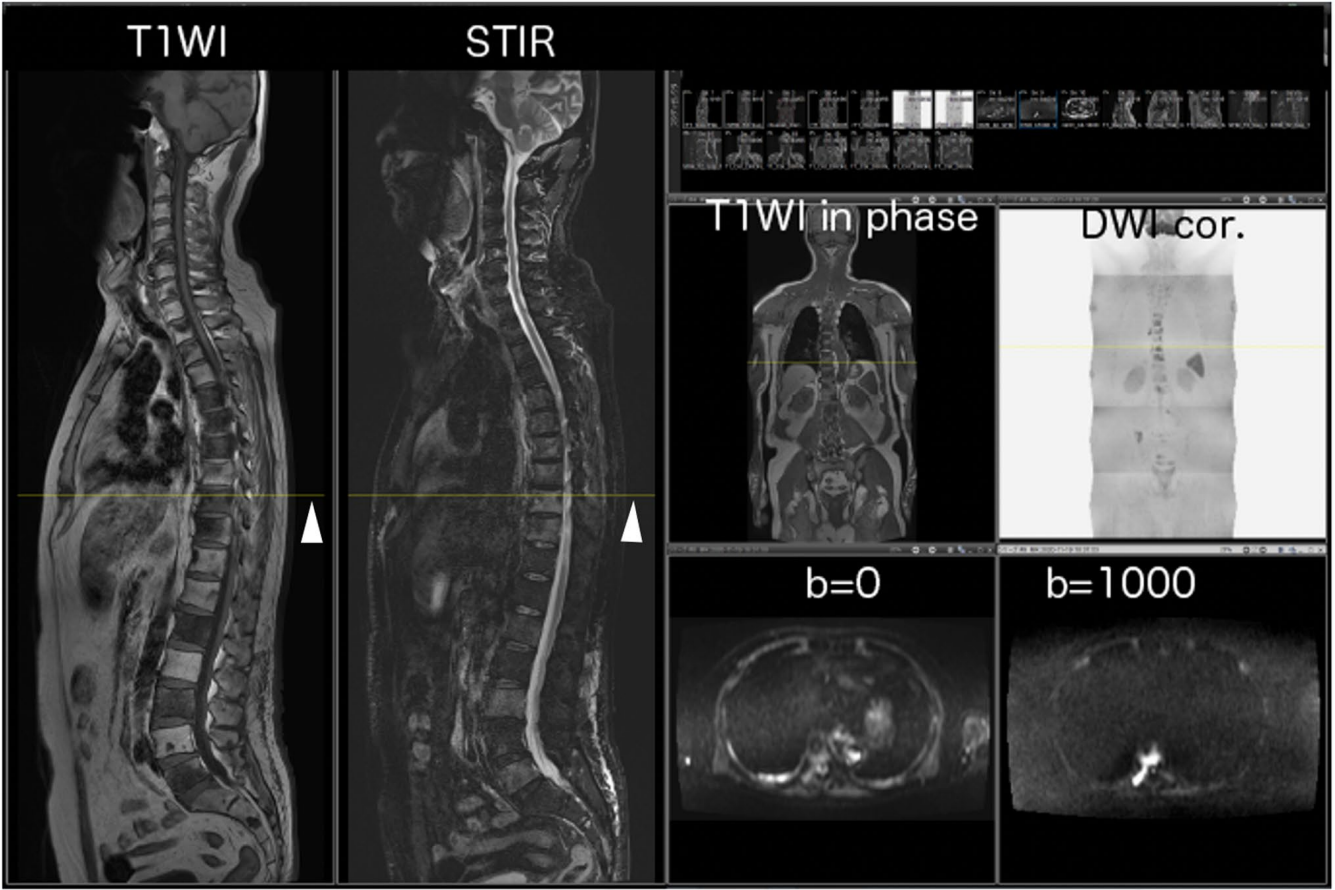
Imaging layout. This image was captured using WB-MRI by the radiologists and comprised two monitors. On the left side of the monitor, the sagittal T1W images and STIR images are displayed by longitudinal two partings, and on the right side of the monitor, the coronal in-phase T1W images, coronal reconstructed DW images, axial *b* = 0, and axial *b* = 1000 images were displayed by four partings. For all image planes, reference lines were used to detect the precise level of the regions (white arrowheads)

The size of the imaging data is enormous; therefore, the environment for image interpretation is important. In our department, two 324.9 × 432.2 mm monitors are used for viewing all station-combined sagittal and coronal direction images simultaneously. Three image planes should always be shown, equipped with a reference line (Fig. 6).

### Tumor quantification

**Fig. 7:**
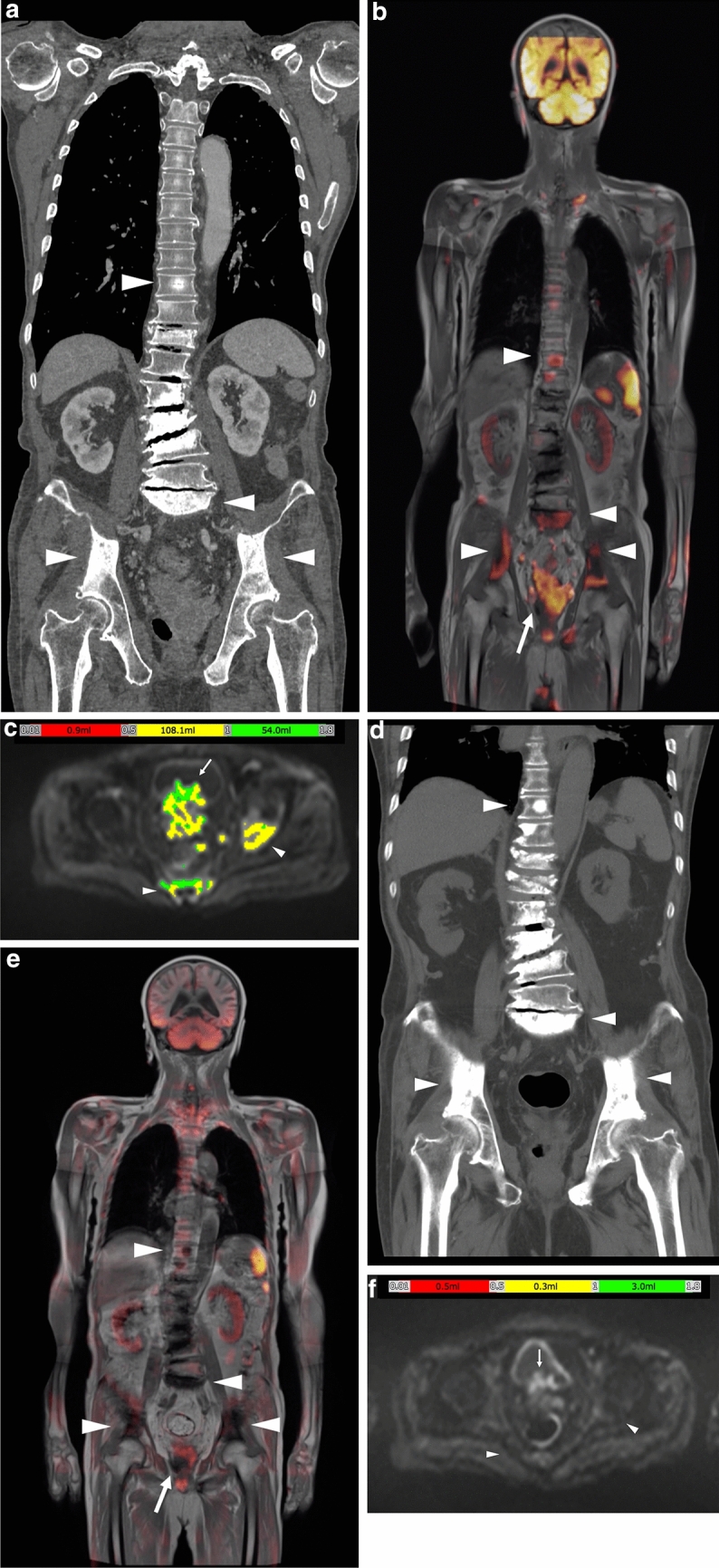
An 85-year-old man who was initially diagnosed with primary unknown multiple bone metastases. **a** On the coronal reconstructed CT image, sclerotic lesions were observed in the bilateral ilium, sacrum and Th10 (white arrowheads), which were considered osteoblastic metastases. Sclerotic change in the lumbar spine appeared to be degenerative. Following the CT examination, a high serum PSA level (> 5000 ng/mL) was detected. **b** On the fused coronal DW and in-phase T1W image at a similar level to **a**, high-intensity areas were shown in the bilateral ilium, sacrum and Th10 (white arrowheads). In addition, the prostate gland was enlarged and appeared as high intensity (white arrow). **c** BD score composed axial image at the level of pelvis. The total volume of the area with a specific ADC value range (0.01 to 1.8 × 10^–3^ mm^2^/s in the image) is defined as tDV and is subcategorized and denoted by a specific color (0.01–05: red, 0.5–1: yellow, 1–1.8: green). Color-displayed lesions are shown in the left acetabulum and sacrum (white arrowheads). The enlarged prostate gland can also be observed as color-displayed lesions (white arrow). In this case, the tDV was 163.0 mL, and the mean ADC of these lesions was calculated as 0.96 (× 10^–3^ mm^2^/s) by the ADC histogram. d-f. Three months after the image shows in **a**–**c**, following combined androgen blockage. PSA decreased to 175.483 ng/mL. **d** On the coronal reconstructed CT, sclerotic change showed an increase from that in **a** in the bilateral ilium sacrum and Th10 (white arrowheads). **e** On the fused image, high-intensity areas in the bilateral ilium, sacrum, and Th10 decreased in intensity (white arrowheads), and the prostate gland decreased in size and intensity (white arrow) from those observed in **b**. **f** On the BD score–composed image, the color-displayed area shown in **c** largely disappeared (white arrowheads and white arrow). tDV markedly decreased to 3.8 mL, and mean ADC value increased to 1.18 (× 10^–3^ mm^2^/s). Osteosclerotic change on **d** had been considered as the re-ossification after therapy

In our department, after obtaining approval from our ethics committee (No18117), we use BD score (PixSpace Inc.), which is the only clinical research tool available and is used only for retrospective analysis. This software was developed for automatically calculating tDV and mean ADC based on the ADC histogram generated using the DWI image and arbitrarily defining the threshold of the ADC value, which ranges from 0 to 3 (× 10^–3^ mm^2^/s). We defined the threshold as 1.8 (× 10^–3^ mm^2^/s), based on the evaluations of the effects of various treatment (66). From all voxel data of *b* = 0 and b = 1000 DWI, lesions in which the ADC value ranges from 0 to 1.8 (× 10^–3^ mm^2^/s) are rapidly extracted. Then the BD score, which is composed of the axial (Fig. 7c) and coronal images, is generated. After that, extracted normal anatomical structures with corresponded ADC value, such as spleen, testis, spinal cord, intestine, etc., are manually removed. Consequently, total diffusion volume of the remaining lesion is defined as tDV and mean ADC value of the area is calculated. The software is particularly useful for processing multiple and large lesions, such as multiple metastatic lesions detected by whole-body DWI. Thus, it has been adopted for evaluating the therapeutic effect of whole-body metastatic lesions.

### Presentations of some typical cases of bone metastases in prostate cancer

WB-MRI is useful not only for diagnosing the distribution and spread of bone metastases, but also for monitoring the therapeutic effect.

In this section, we presented several cases that compare CT findings.

1. Primary osteoblastic metastases (Fig. 2).

In general, osteoblastic-type metastases are frequently observed in prostate cancer in the initial stage. In this case, osteosclerotic change is evident on CT images and appears as low intensity on T1WI images and high intensity on DWI images.

2. Primary mixed-type metastases/inter-trabecular metastases (Fig. 3).

Other types, such as osteolytic and inter-trabecular types, are also detectable.

In this case, CT images do not show the abnormal findings, such as bone formation or destruction, and DWI of MRI show high signal intensity.

3. Primary unknown osteoblastic metastases histologically confirmed as prostate cancer (Fig. 7).

During routine clinical experiences, we often encounter cases of osteoblastic metastases in men with an unknown primary site of origin, and prostate cancer is confirmed following initial radiological examinations. In such cases, multiple high-intensity areas are noted in WB-DWI images, and these are diagnosed as multiple bone metastases. In addition, the prostate gland is carefully observed on axial and coronal reconstructed DWI images. Predicting the primary site before assessing for high serum PSA levels is sometimes possible.

**Fig. 8:**
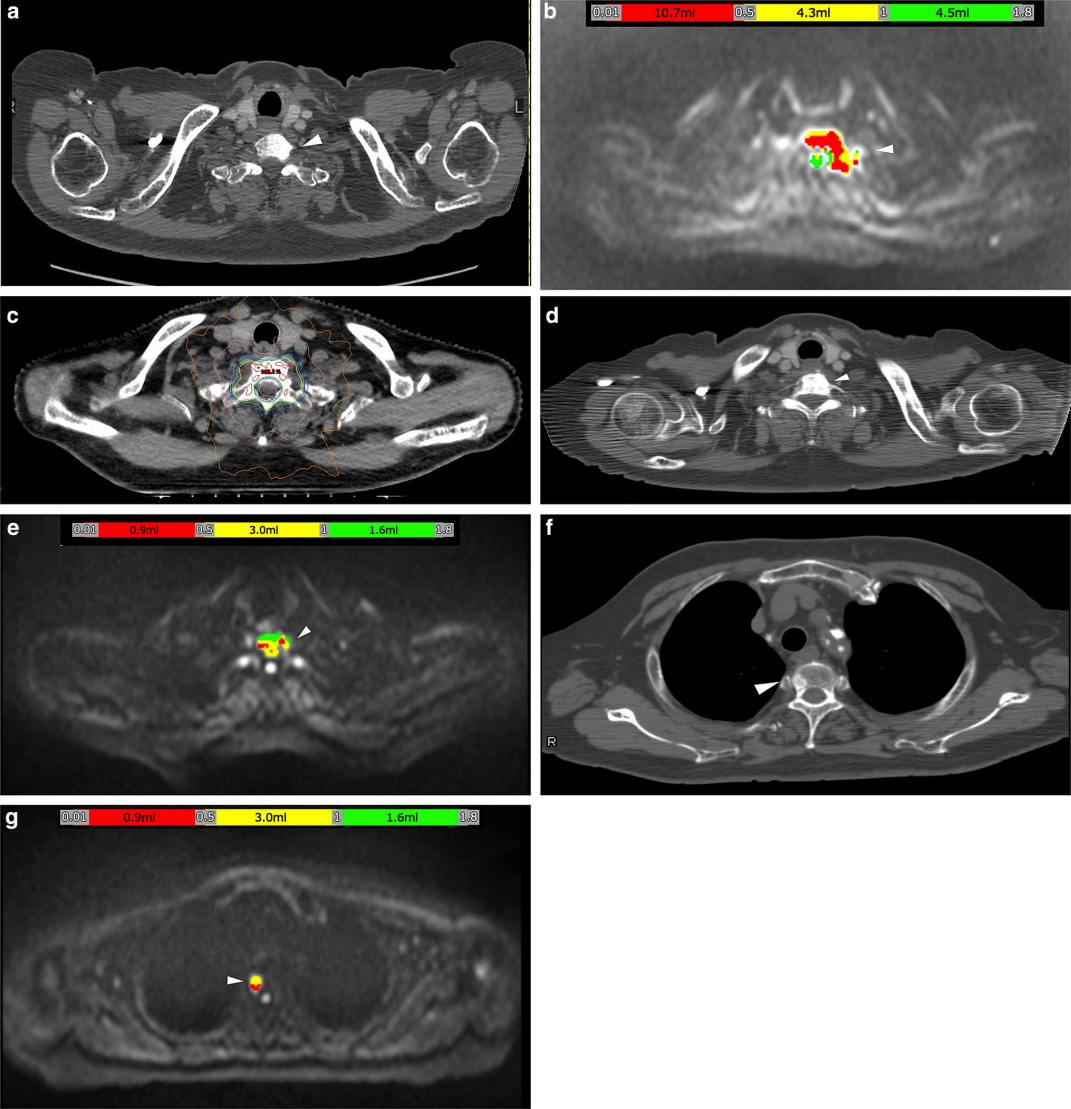
A 70-year-old man with CRPC. Six years ago, local radiotherapy was performed for prostate cancer (T2c N0M0, PSA 32.1 ng/mL, GS 4 + 5). Four years later, the patient’s condition transitioned to CRPC. **a** On the original axial CT at the level of C7, osteosclerotic change was seen in the left part of C7 body (white arrowhead). At that time, the patient’s PSA level was 4.413 ng/mL. **b** On the BD score–composed axial image at the level of C7, a colored area was seen (white arrowhead). tDV was 19.5 mL, and the mean ADC value was 0.74 (× 10^–3^ mm^2^/s). **c** The C7 lesion was regarded a metastatic lesion, intensity-modulated radiation therapy was performed (35 Gy/5fr RT.) with reference to the axial DW image. **d**–**g** Approximately 10 months after the image shown in **a**–**c**, PSA increased to 6.235 ng/mL. **d** On the original axial CT at the level of C7, the osteosclerotic change increased in the left part of the C7 body (white arrowhead) from that shown in **a**. **e** On the BD score–composed axial image at the level of C7, a colored area was seen (white arrowhead). The colored area decreased from that in **b**. **f** On the CT image at the level of Th3, a subtle sclerotic change was newly observed (white arrowhead), but this was a retrospective finding. **g** On the BD score–composed axial image at the level of Th3, a colored area was newly observed in the right part of the body (white arrowhead). In this case, tDV decreased to 5.5 mL, and consequently, mean ADC increased to 0.83 (× 10^–3^ mm^2^/s); however, Th3 was diagnosed as a new metastatic lesion

4. Therapeutic effect of bone metastases (Figs. 7 and 8).

For monitoring the therapeutic effect, both therapy-reactive lesions and newly recurred lesions are often observed in the same case.

DWI enables precise evaluation of such lesions.

Radiologists should be aware that a CT finding of osteosclerotic change in metastatic lesions has two possibilities (Figs. 7 and 8). One of these is the osteoblastic metastasis itself (Fig. 7a and 8f), and the other is the re-ossification of the therapeutic effect (Fig. 7d and 8d). Several previous studies have reported the re-ossification of bone metastases after radiotherapy [70, 71], which mainly describe the improvement in the stability of osteolytic metastases. However, careful differentiation from osteoblastic metastasis is important.

Differentiate on CT is often difficult, whereas DWI allows easy differentiation between these two conditions. The former shows high intensity, and the latter shows decreased signal intensity or tumor volume (Fig. 8b and e). Differentiating the two conditions using only conventional MRI sequence only or CT is difficult.

**Fig. 9:**
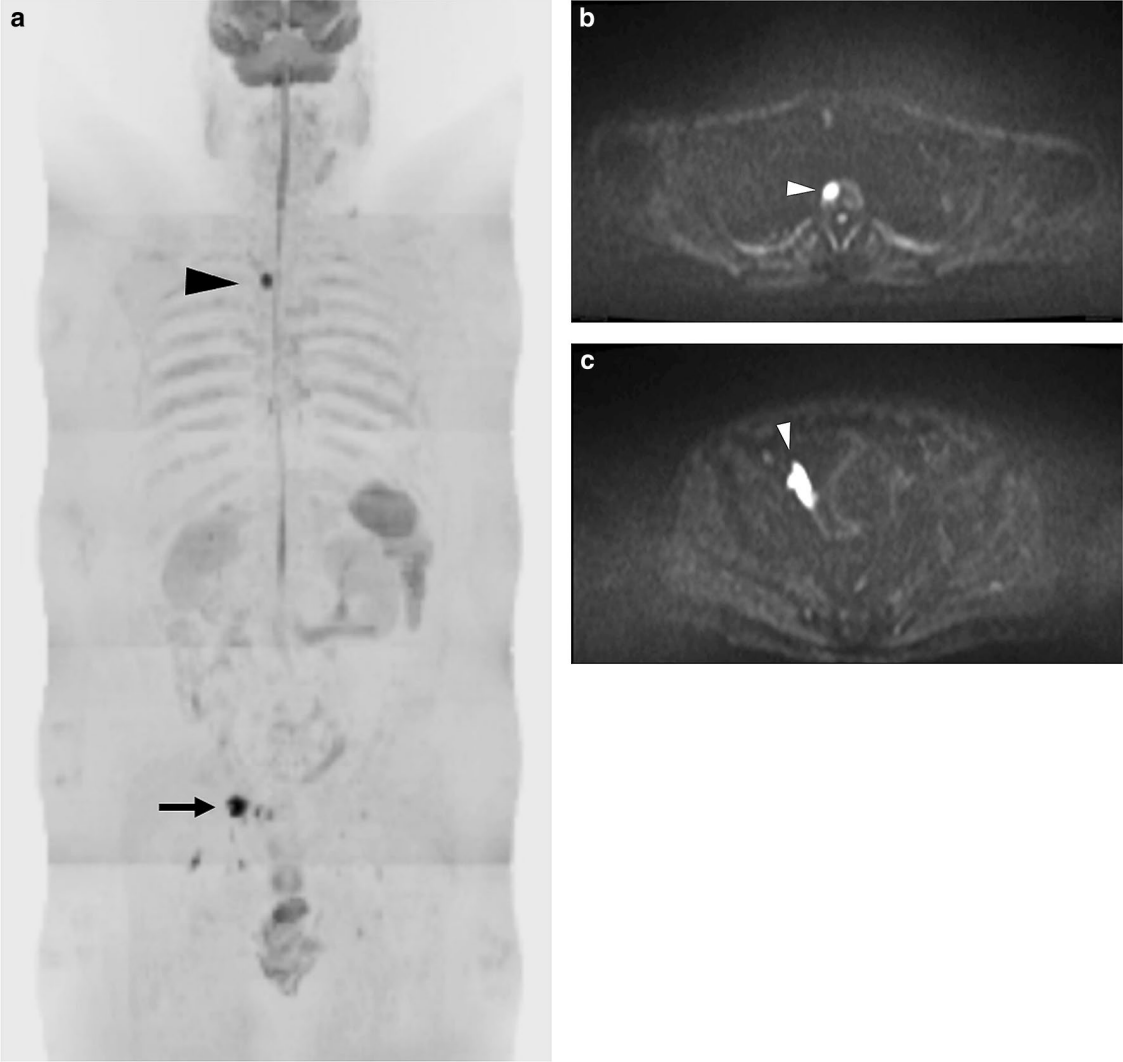
Oligometastases. An 82-year-old man with bone metastases from prostate cancer. **a** On the radial reconstructed antero-posterior DW image, a high-intensity area was observed in Th2 (arrowhead), and swelling of the right internal iliac lymph node was suspected (arrow). At this time, the serum PSA was 34.903 ng/mL. **b** On the *b* = 1000 axial DW image at the level of the Th2, a high-intensity area was seen (white arrowhead). **c** On the *b* = 1000 axial DW image at the level of the pelvis, the right iliac lymph node was swollen and had shown high intensity (white arrowhead)

5. Oligometastases (Fig. 9).

The term “oligometastases” were conceived by Hellman and Weichselbaum in 1995 [72]. They described it as an intermediate state of distant spread, reflecting disease with a low, slow and late metastatic spreading capacity [73, 74]. The exact lesion number or volume is not defined, but the St Gallen Advanced Prostate Cancer Consensus Conference (APCCC) reached a consensus (85% of the panel) that the presence of three synchronous metastases (bone and/or lymph nodes) should be used to define oligo-metastatic prostate cancer [75–77]. Various reports have indicated that the accurate assessment of metastatic burden using radiologic and functional imaging technique is crucial [77]. However, conventional imaging modalities, which include CT findings, have low sensitivity in detecting small-volume disease and may underestimate disease burden [73]. Therefore, WB-MRI has been offered as a modality for effectively detecting oligo-metastases [74, 75, 78, 79].

### Future evolution of WB-MRI

#### Use of artificial intelligence (AI)

AI has been mainly adopted to reduce scanning time [80].

Kidoh et al. [81] conducted a trial of deep learning-based noise reduction for brain MRI. In addition, Kashiwagi et al. [82] developed deep learning-based reconstruction for de-noising brain, lumbar spine, and knee images, with the ultimate goal of shorting scanning time and reduce noise.

Zormpas-Petridis et al. [83] reported a method of using AI with WB-DWI. Their study aimed to improve the image quality of repeated acquisition (NEX) to one image and considerably reduce scanning times and found that image quality was improved and the acquisition time was reduced from 30 to 5 min.

In addition, they indicated that the automatic segmentation of lesions and the removal of normal structures are required for AI when the tDV and mean ADC are calculated.

#### Collaboration of modern radionuclide tracer

Recently, numerous researchers have reported the use of modern radiotracers such as ^11^C-Choline [84, 85] ^18^F-NAF [86, 87], and ^68^ Ga-PSMA-PET [88–91].

^68^ Ga-PSMA-PET has shown to be more accurate than WB-MRI in identifying distant metastases [88, 89]. It is particularly effective in cases where PSA is lower than 0.5 ng/ml [90, 91]. However, the advantage of WB-MRI is its absence of radiation exposure, cost-effectiveness, and examination repeatability. Thus, the combination of WB-MRI and these tracers might be the next trend in research.

## Summary

WB-MRI has been established as the gold standard for detecting bone metastases from prostate cancer. It has the advantages of being able to detect lesions that are overlooked by conventional modalities, such as CT and BS. Moreover, because of its repeatability, it can be used to monitor therapeutic effects. In addition, further shortening of imaging time and automatic image processing will likely continue to progress.

## Data Availability

All cases were belonged to Osaka International Cancer Institute and in all cases, informed consents were acquired. All clinical and imaging data were belonged to Osaka International Cancer Institute.
